# Prediction and Validation of a Druggable Site on Virulence Factor of Drug Resistant *Burkholderia cenocepacia* **

**DOI:** 10.1002/chem.202100252

**Published:** 2021-05-01

**Authors:** Kanhaya Lal, Rafael Bermeo, Jonathan Cramer, Francesca Vasile, Beat Ernst, Anne Imberty, Anna Bernardi, Annabelle Varrot, Laura Belvisi

**Affiliations:** ^1^ Universita' degli Studi di Milano Dipartimento di Chimica via Golgi 19 I-20133 Milano Italy; ^2^ Université Grenoble Alpes CNRS, CERMAV 38000 Grenoble France; ^3^ University of Basel Department of Pharmaceutical Sciences Klingelbergstrasse 50 4056 Basel Switzerland

**Keywords:** Antimicrobial resistance, BC2L-C, Glycomimetics, Ligand design, Virtual screening

## Abstract

*Burkholderia cenocepacia* is an opportunistic Gram‐negative bacterium that causes infections in patients suffering from chronic granulomatous diseases and cystic fibrosis. It displays significant morbidity and mortality due to extreme resistance to almost all clinically useful antibiotics. The bacterial lectin BC2L‐C expressed in *B. cenocepacia* is an interesting drug target involved in bacterial adhesion and subsequent deadly infection to the host. We solved the first high resolution crystal structure of the *apo* form of the lectin N‐terminal domain (BC2L‐C‐nt) and compared it with the ones complexed with carbohydrate ligands. Virtual screening of a small fragment library identified potential hits predicted to bind in the vicinity of the fucose binding site. A series of biophysical techniques and X‐ray crystallographic screening were employed to validate the interaction of the hits with the protein domain. The X‐ray structure of BC2L‐C‐nt complexed with one of the identified active fragments confirmed the ability of the site computationally identified to host drug‐like fragments. The fragment affinity could be determined by titration microcalorimetry. These structure‐based strategies further provide an opportunity to elaborate the fragments into high affinity anti‐adhesive glycomimetics, as therapeutic agents against *B. cenocepacia*.

## Graphical abstract

**A new druggable site for anti‐adhesive therapy**. We used fragment‐based virtual screening to explore the druggability of a region in the vicinity of the fucose binding site in a lectin from an opportunistic and highly drug‐resistant pathogen. The crystal structure of the target protein complexed with the lead fragment validated the existence of a secondary binding site, paving the way for the design of potent ligands to be employed in anti‐adhesive therapy.

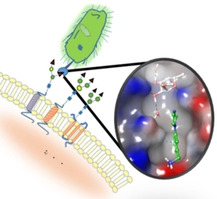

## Introduction

Antimicrobial resistance enables pathogens to resist to the effects of an antibiotic or drug that would usually kill them or limit their growth.^[1]^ The emergence and spread of multidrug‐resistant bacteria have challenged the existing treatment regimen which has enormous implications for worldwide healthcare delivery and community health.[^1^, ^2^] *Burkholderia cenocepacia* is a Gram‐negative bacterium belonging to a group of more than 20 species called *Burkholderia cepacia* complex (BCC).^[3]^ BCC species survive in natural sources including water, soil and vegetation. In Nature, BCC bacteria can have both beneficial and detrimental effects on plants^[4]^ but they are also identified as opportunistic human pathogens. In particular, *B. cenocepacia* is responsible for deadly infections in patients with immunocompromised conditions like chronic granulomatous diseases^[5]^ and cystic fibrosis.^[6]^ The treatment of the infection is really challenging, as *B. cenocepacia* strains show extreme resistance to almost all clinically useful antibiotics^[7]^ and cause significant morbidity and mortality*. B. cenocepacia* produces a large number of virulence factors that play an important role in host cell infection.^[8]^ Among them, four soluble lectins (BC2L‐A, ‐B, ‐C and ‐D) have been identified, displaying very high sequence similarity with the virulence factor LecB (PA‐IIL) from *Pseudomonas aeruginosa*.^[9]^ LecB forms a tetramer with high affinity for fucose,^[10]^ while BC2L‐A is a dimer with significant affinity for mannose and oligomannose‐type N‐glycans.[^9^, ^11^] Except BC2L‐A, the other three *B. cenocepacia* lectins present additional N‐terminal domains.[^11^, ^12^] For BC2L‐C, the C‐terminal domain (LecB like) specifically binds to mannose, while the N‐terminal domain (BC2L‐C‐nt) has been structurally characterized as a novel fucose‐binding domain with a trimeric TNF‐α‐like architecture.^[13]^ Thus, BC2L‐C represents a novel type of superlectin with dual specificity for fucose and mannose in the N‐ and C‐terminal domains, respectively.^[14]^ BC2L‐C as a virulence factor binds to carbohydrates present on the epithelial cells of the host. BC2L‐C‐nt has higher affinity for fucosylated oligosaccharides and its complexes with H‐type 1 and Globo H (H‐type 3) oligosaccharides have been recently solved.^[15]^ The super lectin is proposed to be involved in adhesion and inflammation processes.^[14]^ Bacterial adhesion represents the first step of infection, it also enables bacteria to have access to nutrients and to better resist to immune factors, bacteriolytic enzymes and antibiotics.^[16]^ Therefore, preventing glycoconjugate‐lectin interactions by anti‐adhesive therapy can counteract the infection process at its initial stage.[^16^, ^17^] This inhibition can be achieved by means of carbohydrate‐based synthetic molecules which can compete for the lectin working as antagonists and, thus reduce the level of infection.

Here we describe the development of a structure‐based approach to the design of such antagonists. First, we solved the crystal structure of apo BC2L‐C‐nt and compared the protein surface and bound water molecules with the fucose‐bound structure. The X‐ray crystal structure in complex with methylseleno‐α‐L‐fuco‐pyranoside (MeSe‐α‐L‐Fuc, PDB code 2WQ4) was then used for virtual screening of a small fragment library in the vicinity of the fucose‐binding site. This procedure identified a region (region ‘X’) that was most likely to host potential hits. The results were analysed with the main objective of identifying suitable fragments that docked in region X and could be chemically connected to the fucose core to obtain high‐affinity ligands. The interaction of the fragments with the protein domain was confirmed using a group of biophysical techniques including STD‐NMR.^[18]^ ITC and X‐ray crystallography performed on one fragment confirmed binding at the expected location and therefore the ability of site X to host drug‐like fragments. This study provides the rational design tools to elaborate the selected fragments into high‐affinity ligands.

## Results and Discussion

### Analysis of the binding site in crystal structures

**Figure 1:**
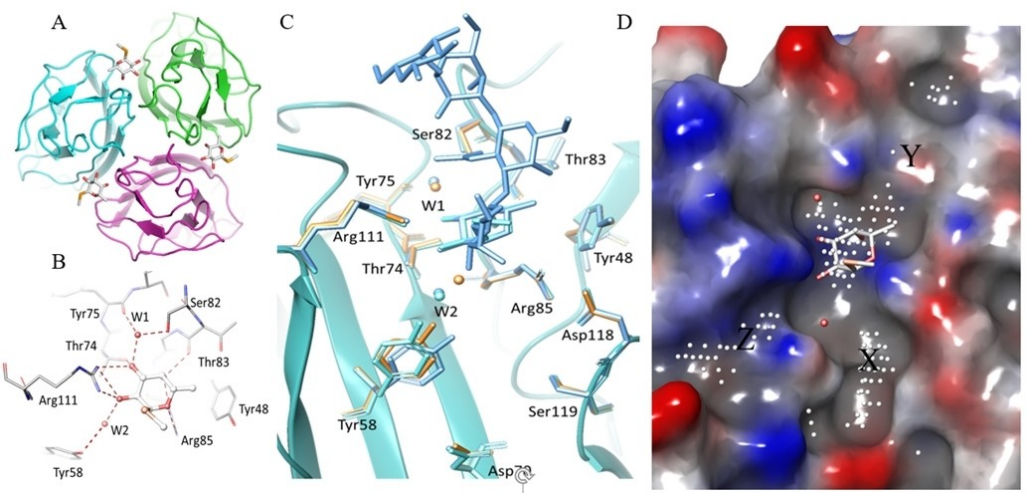
A) Crystal structure of BC2L‐C N‐terminal domain (PDB 2WQ4) showing three identical fucoside binding sites at the interface of monomers B) Fucoside binding site with MeSe‐α‐L‐Fuc. Hydrogen bonds are represented as dashed lines. C) Superimposition of binding sites in the apo (orange, PDB 7BFY) and the holo forms (Cyan PDB 2WQ4, azure PDB 6TIG) of BC2L‐C N‐terminal domain. D) Identification of additional regions (site points) near fucoside binding site suitable for fragment binding.

Crystal structure of trimeric BC2L‐C‐nt complexed with H‐type 1 and Globo‐H oligosaccharides are available[^14^, ^15^] revealing three sugar binding sites located at the interface between neighbouring chains (A, B, C), and separated by a distance of ∼20 Å (Figure 1A). In each fucose binding site (Figure 1B), the key residues Tyr48, Ser82, Thr83, Arg85 from one chain (e. g. chain A) and Tyr58, Thr74, Tyr75, Arg111 from the neighbouring chain (e. g. chain C) play an important role in ligand binding. In addition, two water molecules bridge the sugar and the protein. Both water molecules are conserved in the available X‐ray structures of BC2L‐C‐nt in complex with fucoside and fucosylated oligosaccharides.[^14^, ^15^] One is deeply buried in the binding site and sandwiched between the protein and the ligand, forming an H‐bonding interaction with the HO‐3 of fucose (Figure 1B). The second water molecule is more exposed to the solvent and mediates an H‐bonding interaction between HO‐2 of fucose and the side chain of Tyr58.

The crystal structures of complexes evidenced some promising pockets on the protein surface near the fucose binding site. The occurrence of such pockets in the apo‐protein needed to be verified and therefore, we solved the crystal structure of the apo form of BC2L‐C‐nt at high resolution (1.5 Å). The asymmetric unit in the P6_3_ space group contains one monomer and crystal symmetry was applied to build the trimer for comparison with other structures. Root‐mean‐squares values of 0.21 Å and 0.24 Å were obtained when comparing with the trimer complexed with MeSe‐α‐L‐Fuc and Globo‐H, respectively. Comparison of binding sites (Figure 1C) did not display significant differences in the amino acid of fucose binding pockets and environment. Only a minor difference is observed at the surface loop (Asn52‐Phe54), that is involved in the interaction with methyl group of N‐acetylgalactosamine (GalNAc) in the complex with Globo‐H (Figure S1). Likewise, small differences in the conformation of the N‐ and C‐terminal residues were noticed due to H‐bond interaction between them. The changes in the conformation of the termini further caused a small displacement (0.6 to 1.0 Å) of surface loops (Val28‐Asp35, Asp95‐Val100). Analysis of water molecules involved in bridging fucose to protein indicated that the more buried one (W1) is conserved in all structures, while the more exposed one (W2) moves by 1.9 Å in the apo structure.

The new crystal structure therefore confirms that the region surrounding the fucose binding site is of interest for drug design. This surface was analysed for druggability (ligandability)^[19]^ using the SiteMap^[20]^ tool. SiteMap creates a grid of points based on the depth, size, van der Waals interaction energy, hydrophilicity and hydrophobicity to determine the druggability of a protein region. Based on these characteristics, a single scoring function called SiteScore is assigned to potential druggable regions. For BC2L‐C‐nt the calculations identified three regions, which we labelled X and Y and Z (Figure 1D) in the vicinity of the fucose. Region Y, consisting of residues Ser82, Thr83 and Phe54 in each monomer, corresponds to the area where larger, fucosylated oligosaccharides were observed to bind, including the recently described Globo H hexasaccharide and H‐type 1 tetrasaccharide.^[15]^ Of the two other regions (X and Z), site X is a deep crevice extending along the binding interface. The site Z consists of the region between Val110 and Arg111. All the sites are worth exploring further, thus the docking protocol was built to include them in the analysis.

### Identification of top‐ranked fragments

#### Docking analysis

2000 molecular fragments were retrieved from the Maybridge library of small fragments (rule of 3 diversity set available at https://www.maybridge.com/). In the first docking model, all the fragments were docked in the presence of the two conserved water molecules and the MeSe‐α‐L‐Fuc. In the second docking model, only the buried water molecule and the ligand were retained since the second water molecule is close to region X and rather exposed to the solvent. This second model allowed us to examine the fragments that might be able to replace it. Fragments were found to dock mainly in regions X and Y. The region Y forms a very shallow and exposed binding site, which mostly hosted lipophilic fragments on the surface. Similarly, region Z also hosted a few hydrophilic fragments on the shallow surface. Region X is comparatively deeper and fragments appear to be nestling in it, generating some specific interactions. Therefore, we focused our further efforts on this region.

**Figure 2:**
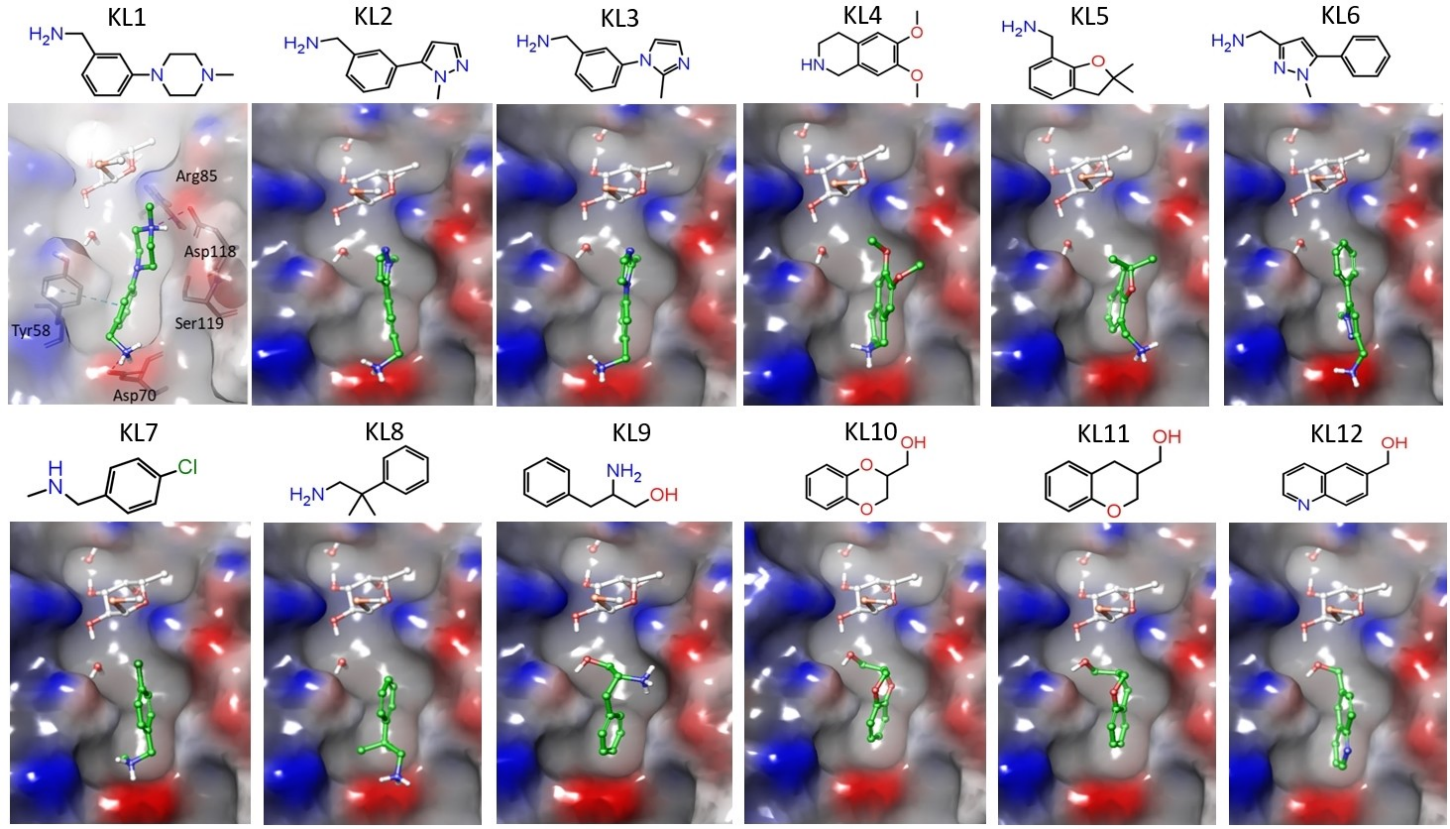
Binding pose for the top ranked fragments (KL1‐KL12) predicted by docking studies at site X. The key residues identified in the binding site are shown in the docking pose of KL1.

Binding analysis of top 200 fragments was done for 6 docking runs with XP, SP and HTVS protocols^[21]^ and involving either one water or two water molecules. HTVS and SP use the same scoring function but the HTVS protocol reduces the number of intermediate conformations, torsional refinement and sampling. The XP protocol employs a different, more complex scoring function with greater requirements for ligand‐receptor shape complementarity. This screens out false positives that SP or HTVS may let through. From each model, the best fragments with consensus scoring (ranked within top 200 fragments) obtained by XP, SP and HTVS were selected for analysis of key residues involved in ligand binding. The docking results with the two waters model showed that the number of hits obtained at site X using SP and HTVS methods were almost same, while the hits obtained using XP were reduced to half. In the one water model, the number of hits at site X increased almost by a factor of two, due to the omitted water molecule near site X. The interaction pattern identified using three scoring functions at site X indicated that the fragments including a benzylamine moiety have good binding affinity. The key residues involved in binding are Tyr58, and Asp70 whilst Asp118 from neighbouring protomer can also be recognized by some of the top scoring fragments that form a salt bridge interaction with it (Figure 2).

The main interactions observed for the majority of the top ranked fragments are a salt bridge between Asp70 side chain and the benzylamino group of the fragments and π‐π stacking interactions with Tyr58. A total of 94 and 89 fragments for site X were identified for one and two waters models, respectively, as top ranked fragments according to XP and SP/HTVS or all the three scoring functions.

#### Selection of best fragments

The fragments were carefully analysed based on different parameters such as structural diversity, possibility to connect them to the fucose core, size and distance from the fucose core.

Small fragments which were found significantly far (>6 Å) from the fucose core and docked on the shallow surface surrounding site X were discarded. The remaining 32 fragments for site X were redocked to analyse the stability of the ligand interactions in multiple binding poses (10 poses). Other factors like commercial availability, synthetic feasibility and purchasing cost allowed to select 12 fragments (Figure 2 and Table S1) for experimental validation. Within this group, fragments KL1‐8 were among the top scorer in the two waters model, while fragments KL9‐12 were predicted to bind in the one water model.

### Experimental validation of fragment binding

For each fragment, a 2.5 mM solution was used to test the interaction with BC2L‐C‐nt using thermal shift assay (TSA, ThermoFluor).^[22]^ Methyl α‐L‐fucoside (Me‐α‐L‐Fuc) was used as a reference in the experiment to observe fucose binding and hence validate the protocol. Then, the fragments were tested in the presence of Me‐α‐L‐Fuc (20 mM). The results show the expected positive shift (∼2 °C) upon Me‐α‐L‐Fuc binding (Figure S2) while all of the complexes with fragments exhibit a small negative shift between 0.15 to 1.65 °C (Figure S3) in the melting temperature (*T_m_*), which possibly suggests that the fragments destabilize the binding interface and bind to a non‐native or partially unfolded state of the protein.^[23]^

We repeated the experiment for all the fragments in the absence of Me‐α‐L‐Fuc and the results show similar behaviour with a smaller negative shift in the melting temperature (Figure S4). The experimental results of TSA do not afford any structural information concerning the interaction. Therefore, we performed another screening using STD‐NMR and X‐ray crystallography.

### STD‐NMR analysis of fragment binding

Saturation transfer difference (STD) NMR has become a leading technique to characterize fragment−macromolecule interaction in solution, because it is sensitive to weak binding events (dissociation constant in a low μM to mM range).[^18^, ^24^] In general, STD experiments are performed by irradiating the methyl group of valine, leucine, or isoleucine residues (between 1 and −1 ppm), that are often present in the binding site of proteins.^[18]^

The irradiation frequency of STD can also be varied in order to investigate whether the fragment has a preferred interaction with aliphatic or aromatic amino acids of the protein.^[25]^

**Figure 3:**
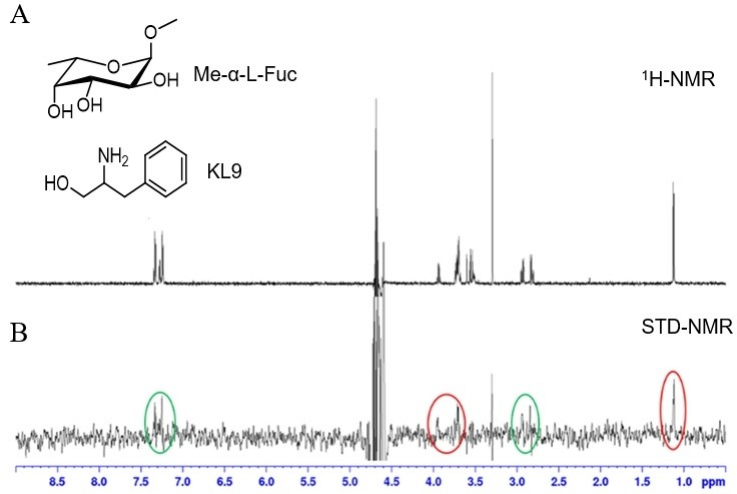
A) 1H‐NMR and B) STD spectrum of fragment KL9 and Me‐α‐L‐Fuc in the presence of BC2L‐C‐nt (1000 : 1) recorded with a Bruker Avance 600 MHz spectrometer. The spectrum is recorded at 298 K with irradiation frequency at −0.05 ppm. In the STD spectrum, the signals at 3.7 ppm and 1.1 ppm, produced respectively by the fucose ring and by its methyl group, are highlighted with red circles. The signals of the fragment are highlighted with a green circle (at 2.9 ppm for −CH2‐Ph and 7.3 ppm for the aromatic protons.

**Figure 4:**
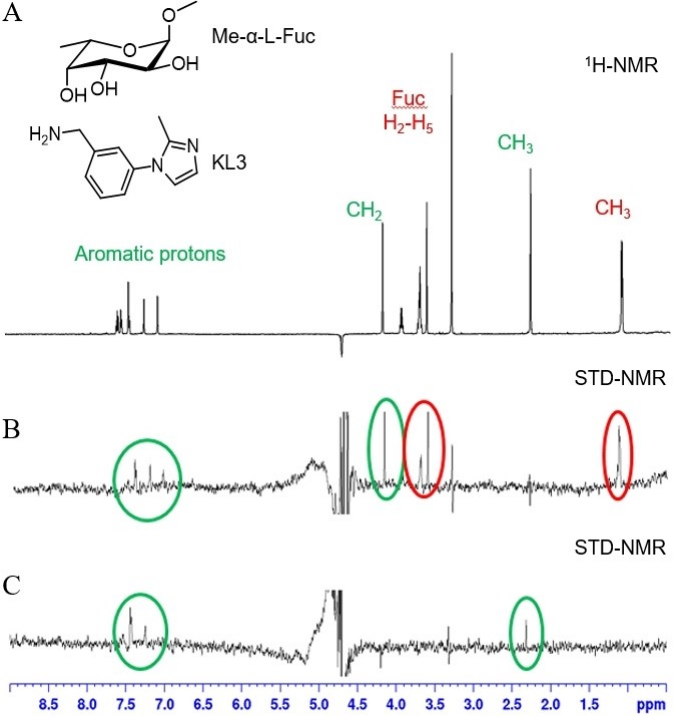
A) ^1^H‐NMR spectrum, B) STD spectrum (irradiation frequency −0.05 ppm) and C) STD spectrum (irradiation frequency 10 ppm) of fragment KL3 and Me‐α‐L‐Fuc in the presence of BC2L‐C‐nt (1000 : 1) recorded with a Bruker Avance 600 MHz spectrometer at 298 K. In the STD spectrum at −0.05 ppm (B), the signals at 3.7 ppm and 1.1 ppm, produced respectively by the fucose ring and by its methyl group, are highlighted with red circles. The signals of the fragment are highlighted with a green circle (at 4.2 ppm for −CH_2_− and in the range 7.05–7.4 ppm for aromatic protons). The STD spectrum at 10 ppm (C) shows the aromatic protons (in the range 7.1–7.4 ppm) and the methyl group (at 2.3 ppm) of the fragment

STD‐NMR was used to analyse the interaction of BC2L‐C‐nt with fragments KL3, KL8 and KL9 in the presence of Me‐α‐L‐Fuc, irradiating at −0.05 ppm. Me‐α‐L‐Fuc was initially tested alone in the experiment, verifying that it binds BC2L‐C‐nt, with a strong involvement of the methyl group (Figure S5). Then, fragment KL3, KL8 (among the top scorers in the two waters docking model) and fragment KL9 (predicted to bind by the one water model) were analysed in the presence of the protein and of 2 mM Me‐α‐L‐Fuc. The sample was prepared at 1 : 1 ratio between sugar ligand and fragment. The resulting spectra for fragment KL9 and KL3 are shown in Figure 3 and Figure 4B, respectively. The spectra of fragment KL8 are reported in the supplementary information (Figure S6). In all cases, simultaneous interaction of the fragment and Me‐α‐L‐Fuc with BC2L‐C‐nt was observed, confirming the binding event for the three fragments in the BC2L‐C‐nt/fucose complex. In the STD spectra, the signals of Me‐α‐L‐Fuc and of the fragment appear with comparable intensities, indicating a similar affinity for sugar and fragment.

STD spectra were also acquired using 10 ppm as irradiation frequency. In this case, the aromatic protons of the fragments are observable, while no signals of Me‐α‐L‐Fuc can be detected (Figures 4C and Figure S7). This finding suggests that the fragments bind in the proximity of aromatic residues of the protein and thus supports the docking prediction that they are located in a protein binding pocket that includes an aromatic residue (Tyr58). It is interesting to note that the STD spectrum of KL3 obtained irradiating at 10 ppm (Figure 4C) also shows a clear signal for the methyl group of the fragment (at 2.3 ppm), which is not visible when irradiating at −0.05 ppm (Figure 4B). This suggests that also this moiety is proximal to an aromatic side chain of the protein. On the contrary, the singlet at 4.2 ppm, corresponding to the methyleneamino benzylic protons of the fragment, which is clearly visible when irradiating at −0.05 ppm (Figure 4B), disappears from the spectrum, like the fucose protons, when irradiating at 10 ppm. Thus, this moiety is expected to be surrounded by aliphatic protons of the protein.

### KL3‐BC2L‐C‐nt crystal structure analysis

All fragments (KL1‐KL12) were soluble enough to be used for soaking experiment with crystals of BC2L‐C‐nt complexed with Globo H hexasaccharide obtained as described previously.^[15]^

**Figure 5:**
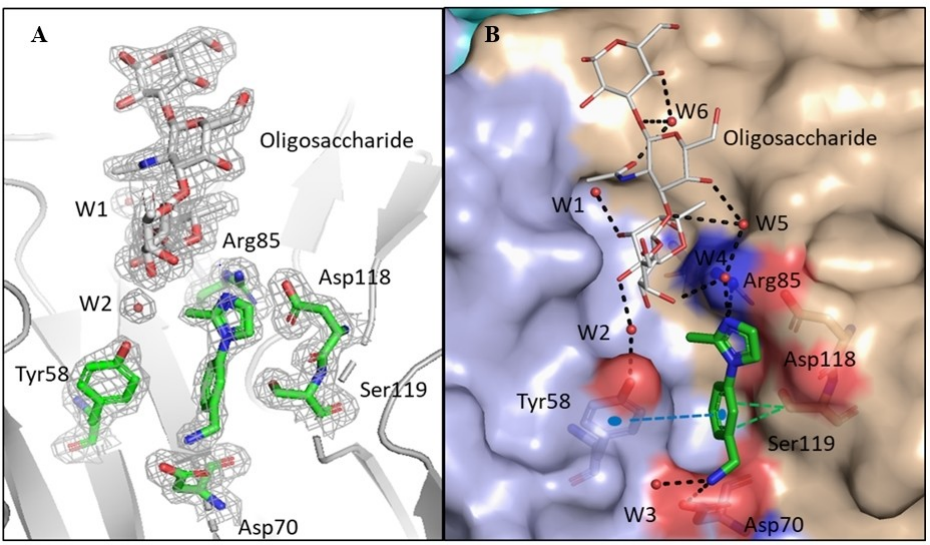
Crystal structure of BC2L‐C‐nt with Globo H and KL3. A) Zoom in the binding site with 2Fo‐DFc electron density represented at 1σ B) Network of interaction in the binding site. Analysis of the complex shows that the key interactions and residues predicted from docking studies were involved in the ligand (KL3) binding. The salt bridge between Asp70 side chain and benzylamino group and π‐π stacking interactions with Tyr58 are maintained in the crystallized complex. In addition to the water molecules from two waters model, a new network of water molecules involved in key interactions between the ligand and the protein is also highlighted. H‐bonding interactions and hydrophobic interactions are displayed in black and green dashed lines respectively. π‐π stacking interactions are shown in blue dashed lines.

After soaking, crystals containing KL10, KL11 and KL12 did not diffract at sufficient resolution for data collection. Crystal soaked with the remaining fragments (KL1‐KL9) diffracted at a resolution close to 2 Å or better, but examination of the electron density after molecular replacement only revealed electron density for the sugar and not for the fragment indicating that they did not bind to the protein in the experimental conditions used. Only in the complex with KL3 (3‐(2‐Methyl‐1H‐imidazol‐1‐yl) benzylamine) at 1.9 Å resolution, electron density corresponding to the expected fragment could be seen in site X located at the interface between two monomers. The orientation of the fragment, and the observed interactions correspond very well with those predicted by the docking studies (Figure 5 and Figure S8). Residue Tyr58 forms T‐shaped π‐π stacking interactions with the benzene ring and Asp70 forms a salt bridge with the amino group in the fragment. The free nitrogen of the imidazole ring makes water mediated interaction with the side chain of Arg85 and the OH‐4 of the GlcNAc moiety of Globo H (Figure 5B). The fragment binds with identical pose and reproduce the same binding interactions in the three binding sites of the trimer (Table 1).

**Table 1:** Summary of the interactions of BC2L‐C‐nt with KL3 in three binding sites.

Ligand atom	Protein or water atom	Distance [Å]
N3	Asp70 (OD2)	3.20
	W3 (HOH161)^[a]^	2.75±0.15
N1	Arg85 (NH2)	3.30±0.07
	W4 (HOH108)	2.46±0.05
C^[b]^	Ser119 (CB)	3.60±0.04
	Tyr58 CE1)	3.50±0.07

[a] only present in two binding sites; [b] For hydrophobic contacts and π‐π interactions, the distance is calculated from the nearest atoms in the ligand and the protein. Mean distance and standard deviation were calculated from the distance of ligand and protein atoms in each binding site.

The position of the fragment in site X is fully consistent with the STD‐NMR data (Figure 4), which indicate proximity of the benzylic methyleneamino group of KL3 to aliphatic residues of the protein (Asp70 and Ser119 in the X‐ray structure). The methyl group of KL3, which in the STD spectra responds to irradiation at 10 ppm, is in fact close to the Tyr58 side chain in the X‐ray structure. The water mediated interactions with Globo H were identical to the previous complex.^[15]^ The results of X‐ray crystallographic screening validated the docking results and the ligandability of site X.

### Affinity analysis and activity validation

The affinity of BC2L‐C‐nt for KL3 was determined by isothermal titration calorimetry (ITC) measurements.^[26]^ Titration of the lectin by KL3 resulted in small exothermic peaks after correction for buffer mismatch (Figure S9) The integrated curve could be fitted with one‐site model with stoichiometry of one, resulting in the determination of a *K*_d_ of 877 μM. Because of the low c‐value of the experiment, the thermodynamic contributions cannot be safely estimated.

## Conclusions

Crystal structure analysis of the apo and the holo form of BC2L‐C‐nt demonstrated the presence of a druggable (ligandable) region (site X) in the vicinity of the fucoside binding site. The computational and experimental screenings identified fragments interacting with BC2L‐C‐nt. The study indicates that the fragments bind in a newly identified binding region in BC2L‐C‐nt when the fucoside binding site is occupied. Different biophysical techniques including TSA and STD‐NMR spectroscopy, confirmed fragment‐protein interaction. Remarkably, the binding mode of one fragment (KL3) could be validated by X‐ray crystallography at high resolution, further confirming the ability of site X to host drug‐like fragments. The affinity measured by ITC is sub‐millimolar, which is very promising for such small fragment. The complementary structural and thermodynamic data give clear view of the relative importance of apolar and polar interactions for fragment KL3. This could be used in the future for structure‐based optimization of this first hit.

Most interestingly, this study provides an opportunity to connect the best fragments to the fucose core to obtain high affinity glycomimetic ligands. The selection of suitable linkers can be done based on the distance (measured 4.8 Å) between the nearest atoms of fragment and the fucose core. Other factors like synthetic feasibility and possibility to maintain the binding pose at the site X can be considered to identify suitable linkers. A robust synthetic route to glycomimetics comprising fucose linked fragments will help in designing high affinity ligands as anti‐adhesive agents against *B. cenocepacia*.

## Experimental Section

**Protein expression and purification**: Protein production and purification of the BC2L‐C‐nt was performed as described previously.^[15]^ An average yield 5.2 mg.L^−1^ of culture medium was obtained and stored at 4 °C.

**Preparation of protein model**: All the calculations were performed using the Schrödinger Suite through Maestro (version 2018‐1) graphical interface.^[27]^ Atomic coordinates from the crystal structure of BC2L‐C‐nt complexed with MeSe‐α‐L‐Fuc (PDB code 2WQ4) were taken from the Protein Data Bank.^[28]^ The asymmetric unit contains three peptide chains and three carbohydrate ligands (MeSe‐α‐L‐Fuc), around a 3‐fold pseudo axis of symmetry. The mode of binding for the sugar is identical in the three binding sites, therefore only one binding site located between chains A and C was used for the calculations. The two structural water molecules HOH2195 (W1) and HOH2194 (W2) bridging fucose and protein were also retained. The hydrogen atoms were added and p*K*_a_ was predicted for protein residues using the PROPKA^[29]^ method at pH 7.4 and assigned HIE protonation state to the histidine (His116) residue. Finally, protein‐ligand complex was subjected to restrained minimization with convergence of heavy atoms to an RMSD of 0.3 Å using the OPLS3 force field.^[30]^

**Preparation of ligand models**: The Maybridge library of small fragments (rule of 3 diversity set) containing 2000 fragments was used for *in silico* screening. The LigPrep^[31]^ tool was used to generate tautomers, stereoisomers and protonation states at pH 7±2. The calculation generated 2904 structures.

**Models for docking study**: For docking grid generation, the centroids of residues from chain A (Tyr48, Ser82, Thr83, Arg85) and chain C (Tyr58, Thr74, Tyr75, Arg111) were selected to define a cubic grid box of 32×32×32 Å. The ligand (MeSe‐α‐L‐Fuc) and the water molecules (HOH2194 and HOH2195) were retained. The same residues were used to generate the second grid with one water (HOH 2195) molecule. Both the grids (models) were used for docking studies using XP, SP and HTVS scoring functions. All the calculations were accomplished by Glide (version 7.8)^[21]^ using the flexible docking approach.

**Thermal shift assay (TSA)**: The fragments KL1, KL2, KL3, KL5, KL6, KL7, KL9, KL10, KL11 (Table S1) were purchased from the Maybridge (Fisher Scientific International) and the other fragments; KL4, KL8 and KL12 were purchased from the abcr GmbH. The fragments were tested for the purity using liquid chromatography‐mass spectrometry (LC–MS).

For the dye‐based TSA, BC2L‐C‐nt (5 μM) in assay buffer (20 mM Tris HCl, 100 mM NaCl, pH 8.0) was incubated with 50x SYPRO orange and 2.5 mM KL1‐12 in the presence or absence of 20 mM Me‐α‐L‐Fuc. A Qiagen Rotor‐Gene Q instrument was used to apply a heat ramp of 1 °C/min from 25–95 °C and SYPRO orange fluorescence at 620 nm was monitored using the appropriate optical channel.

**STD‐NMR interaction studies**: The interaction between ligands and isolated protein was investigated using STD‐NMR experiments. The spectra were acquired with a Bruker Avance 600 MHz instrument at 298 K, in a 3 mm NMR tube and in the phosphate buffer previously described (200 μL). All protein−ligand samples were prepared in a 100 : 1 and 1000 : 1 ligand/protein ratio in concentration. In STD experiments water suppression was achieved by using the WATERGATE 3‐9‐19 pulse sequence. The on‐resonance irradiation of the protein was kept at −0.05 ppm and 10 ppm. Off‐resonance irradiation was applied at 200 ppm, where no protein signals were visible. Selective presaturation of the protein was achieved by a train of Gauss shaped pulses of 49 ms length each. The total length of the saturation train depends on the L7 parameter (the loop counter). STD experiments were acquired with L7=60 leading 2.94 s of total saturation. Two protocols for sample preparation were followed in all cases: either by adding the fragment to a pre‐incubated solution of protein and Me‐α‐L‐Fuc, or by adding the fucoside to a pre‐incubated solution of protein and fragment. The resulting STD spectra were very similar independent of the set up. So, the results reported here correspond to the experiments obtained by adding the fragments to a solution of protein and Me‐α‐L‐Fuc.

**X‐ray crystallography, data collection, and structure determination**: The apo form of BC2L‐C‐nt was crystallized using the vapour diffusion method and 2 μL hanging drops containing a 50 : 50 (v/v) mix of protein (5.5 mg/ml) and reservoir (sodium citrate 1.2 M at pH 7.0). Cubic crystals were obtained from the solution after 3 weeks. For the soaking experiments, crystals of BC2L‐C‐nt in complex with Globo H oligosaccharide were obtained as described previously.^[15]^ The fragments were tested for the aqueous solubility at higher concentration and a stock solution was prepared. The crystals were soaked overnight in the 0.5 μL volume of fragments (from stock) in 4.5 μl of 2.5 M sodium malonate used for cryoprotection that makes a final concentration of 2 mM, for the fragments KL1, KL7 and KL11, 2.5 mM for KL12, 5 mM for KL2, KL5, KL6, KL8 and KL10 and 10 mM for KL3, KL4, KL9. For KL2 and KL12, 10 percent DMSO was added to achieve the above concentration. The crystals were flash‐cooled in liquid nitrogen prior to data collection. The data was collected on the beamline Proxima 1, synchrotron SOLEIL, Saint Aubin, France, using an Eiger 16 m detector (Dectris, Baden, Switzerland). The data was processed using XDS and XDSME.^[32]^ The CCP4 suite was used for all further processing.^[33]^ The coordinates of the monomer A of PDB code 2WQ4 were used as search model to solve the structures of the apo form and the complexes with BC2L‐C‐nt by molecular replacement using PHASER.^[34]^ Refinement was performed using restrained maximum likelihood refinement and REFMAC 5.8^[35]^ interspaced with using manual rebuilding in Coot.^[36]^ for cross validation, 5 % of the data were set aside. Riding atoms were added during refinement (Table S2). Library for the fragment was made using ligand builder in Coot. All carbohydrates were validated using Privateer in CCP4i2 prior validation using the PDB validation server and deposition to the Protein Data Bank under code 7BFY for the apo form and 6ZZW for the complex.

**ITC measurements**: The ITC experiments were performed at 25 °C with an ITC200 isothermal titration calorimeter (Microcal‐Malvern Panalytical, Orsay, France). The protein (BC2L‐C‐nt) and ligand (KL3) were dissolved in the same buffer composed of 100 mM Tris HCl pH 7.0 and 100 mM NaCl. A total of 38 injections of 1 μL of ligand solution (15 mM) were added at intervals of 200 s while stirring at 850 rpm was maintained to ensure proper mixing in the 200 μL sample cell containing the protein, at 225 μM. A control experiment was performed by injecting same concentration of KL3 in buffer. The differences of integrated peaks were performed using the Microcal PEAQ‐ITC analysis software. The binding thermodynamics was further processed with a “one set of sites” fitting model. The experiment determined experiment affinity (*K*_d_), binding enthalpy (▵H) while the stoichiometry was fixed to 1. Free energy change (ΔG) and entropy contributions (TΔS) were derived from the equation ΔG=ΔH−TΔS. The experiments were performed in duplicates and the standard deviation was in 20 % range for *K*_d_.

## Conflict of interest

The authors declare no conflict of interest.

## Supporting information

As a service to our authors and readers, this journal provides supporting information supplied by the authors. Such materials are peer reviewed and may be re‐organized for online delivery, but are not copy‐edited or typeset. Technical support issues arising from supporting information (other than missing files) should be addressed to the authors.

Supplementary
