# Supplementary material for: Prediction and Validation of a Druggable Site on Virulence Factor of Drug Resistant Burkholderia cenocepacia
Source: Chemistry. 2021 May 1;27(40):10341–8. doi: 10.1002/chem.202100252 (PMC8360069; doi:10.1002/chem.202100252)
Supplement: Supplementary file 1 — Supplementary [file CHEM-27-10341-s001.pdf]

Chemistry–A European Journal

Supporting Information

Prediction and Validation of a Druggable Site on Virulence Factor of Drug Resistant *Burkholderia cenocepacia***

Kanhaya Lal, Rafael Bermeo, Jonathan Cramer, Francesca Vasile, Beat Ernst, Anne Imberty,* Anna Bernardi,* Annabelle Varrot,* and Laura Belvisi*

Table S1 Top ranked fragments identified for site X.

Fragment name	Structure	Molecular weight	Aqueous Solubility(mM)
KL1	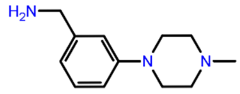	205.3	20
KL2	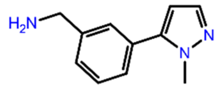	187.2	50[b]
KL3	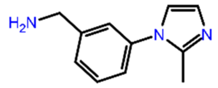	187.2	100
KL4	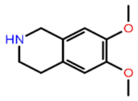	193.2	100
KL5	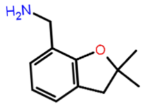	177.2	1[a]
KL6	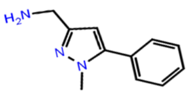	187.2	1[a]
KL7	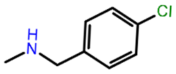	155.6	20
KL8	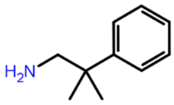	149.2	50
KL9	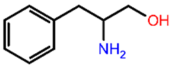	151.2	100
KL10	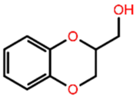	166.2	1[a]
KL11	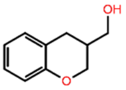	164.2	20
KL12	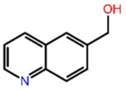	159.2	25[b]

[a] Solubility at higher concentration is not known

[b] dissolved in 10 percent DMSO

Table S2 X-ray data collection and processing of apo form of BC2L-C-nt and its complex with Globo H and fragment KL3.

Data set	BC2L-C-nt complex with KL3 and Globo H			BC2L-C-nt apo form
PDB code	6ZZW			7BFY
**Data Collection**				
Beamline	PROXIMA1 (SOLEIL)			PROXIMA1 (SOLEIL)
Wavelength (Å)	0.9786			0.9801
Space Group	C2			$P6_3$
a, b, c (Å)	74.46, 42.91, 103.34			42.99, 42.99, 94.68
α, β, γ (°)	90.0, 96.10, 90.0			90.0, 90.0, 120.0
Resolution (Å)[a]	37.13-1.90 (1.94-1.90)			19.97-1.50 (1.53-1.50)
Total observations	175918			261006
Unique reflections	25522			15915
Multiplicity[a]	6.9 (7.1)			16.4 (15.5)
Mean $I/\sigma(I)$[a]	10.2(4.0)			21.7 (5.9)
Completeness (%)[a]	98.8 (98.1)			99.9 (100)
R_{merge}[a,b]	0.13 (0.53)			0.081 (0.445)
R_{pim}				0.030 (0.274)
$CC_{½}$[a,c]	0.99 (0.89)			0.999 (0.935)
**Refinement**				
Reflections: working/free[d]	24288 / 1224			15120 / 762
R_{work}/ R_{free}[e]	0.181 / 0.238			0.149 / 0.178
Ramachandran plot: allowed/favoured/outliers (%)	100 / 97 / 0			100 / 97 / 0
R.m.s. bond deviations (Å)	0.014			0.014
R.m.s. angle deviations (°)	1.840			1.855
R.m.s. chiral deviations	0.093			0.085
No. atoms / Mean *B*-factors (Å^2)	Chain A	Chain B	Chain C	Chain A
Protein	962 / 22.1	971 / 22.2	951 / 21.3	952 / 15.6
carbohydrate ligand	58 / 36.1	58 / 33.3	47 / 30.6	-
ligand[f]	14 /32.0	14 / 38.7	14 / 36.0	-
water	69 / 26.9	65 / 26.6	72 / 24.9	150 / 28.2

[a] Values for the outer resolution shell are given in parentheses.

[b] $R_{merge} = \sum_{hkl} \sum_i |I_i(hkl) - \langle I(hkl)\rangle| / \sum_{hkl} \sum_i I_i(hkl)$.

[c] $CC_{½}$ is the correlation coefficient between symmetry-related intensities taken from random halves of the dataset.

[d] The data set was split into "working" and "free" sets consisting of 95 and 5% of the data, respectively. The free set was not used for refinement.

[e] The R-factors R_{work} and R_{free} are calculated as follows: $R = \sum(| F_{obs} - F_{calc} |)/\sum| F_{obs} |$, where F_{obs} and F_{calc} are the observed and calculated structure

factor amplitudes, respectively

[f] refers to ligands bound in the active site and potential surface binding sites

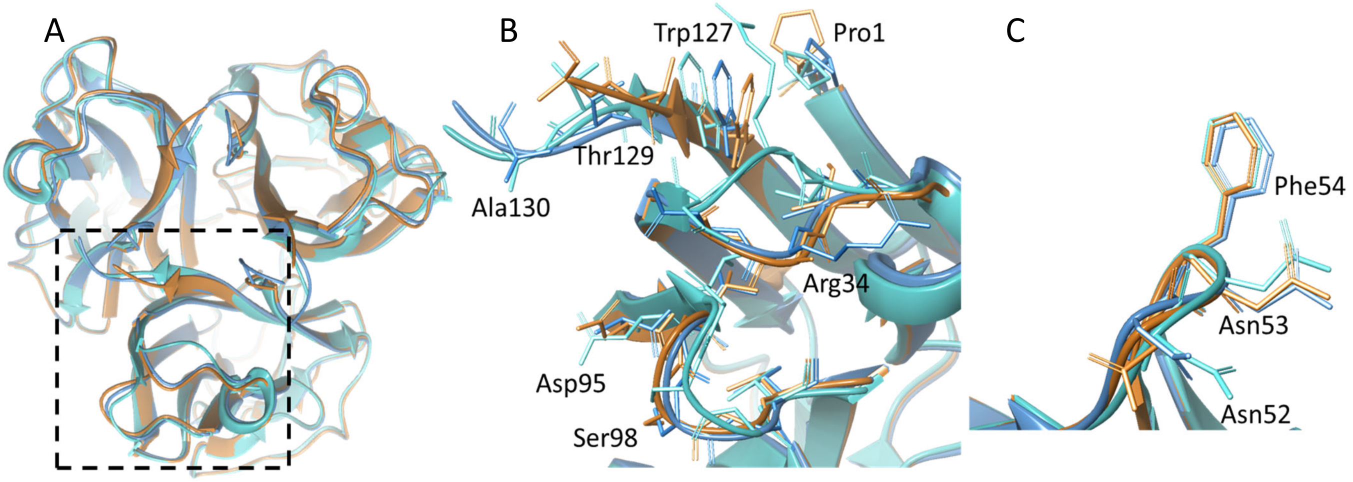

Figure S1 A) Overview of the surface loops in the lower face of BC2L-C-nt holo (cyan (2WQ4) and azure (6TIG)) and apo forms (orange (7BFY)). B) Close-up view of the loop conformation changes observed in both forms. C) Minor differences observed in the loop (Asn52-Phe54) near oligosaccharide binding site.

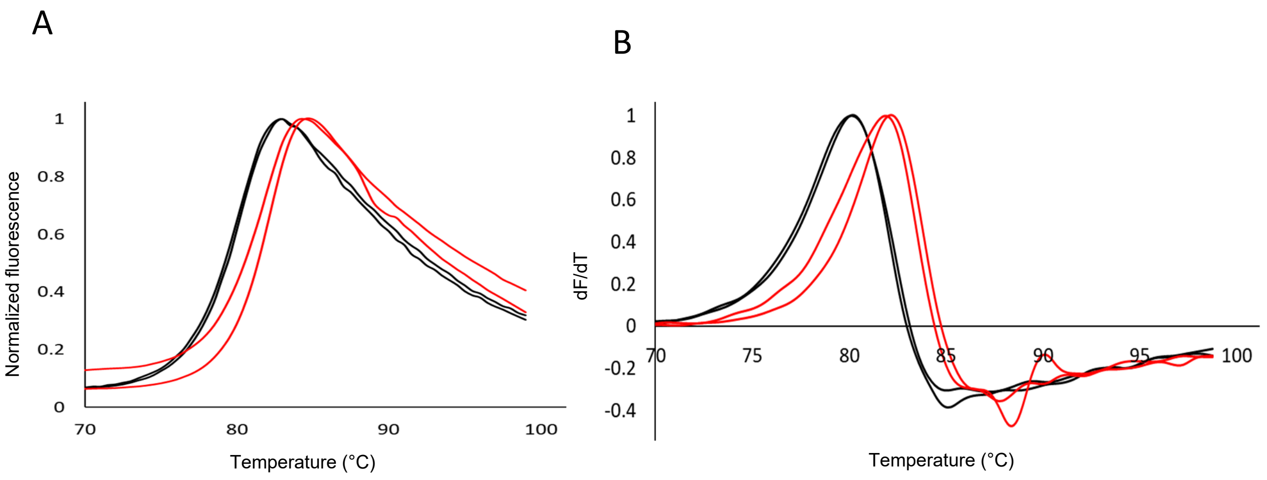

Figure S2 A) Methyl α-L-fucoside (20 mM) binding studies showed a positive shift in the melting temperature (*Tm*) of BC2L-C-nt in the presence (red) and absence (black) of methyl α-L-fucoside. B) First derivatives of fluorescence curves.

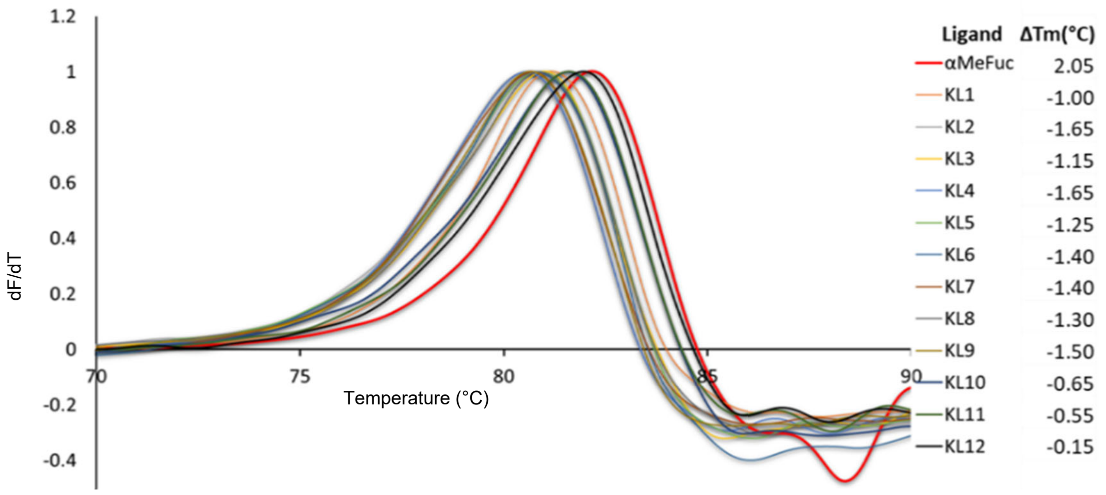

Figure S3 First derivatives of fluorescence curves of the fragments (KL1-12) in the presence of methyl α-L-fucoside (20 mM). Fragments KL1-12 causes a negative shift in the melting temperature (*Tm*) of BC2L-C-nt which indicates ligand interaction.

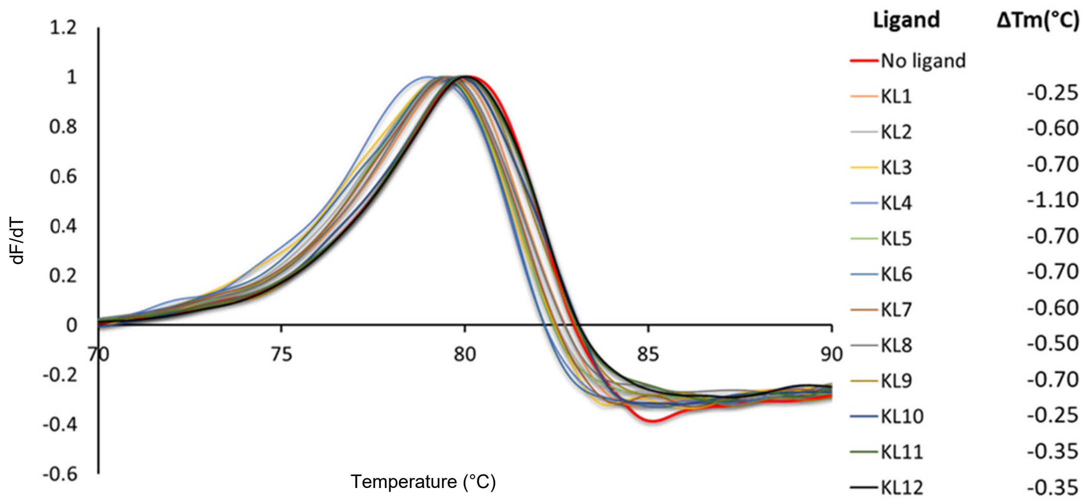

Figure S4 First derivatives of fluorescence curves of the fragments (KL1-12) in the absence of methyl α-L-fucoside. Fragments KL1-12 show a negative shift in the melting temperature (*Tm*) of the protein which indicates ligand interaction.

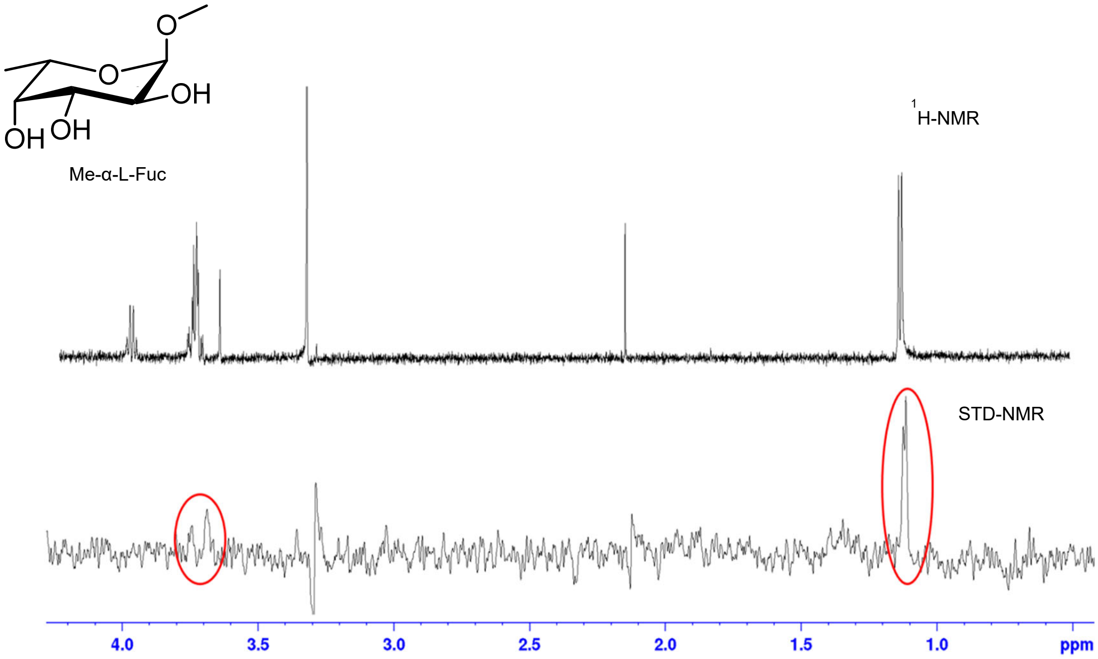

Figure S5 ^{1}H-NMR (upper) and STD spectrum (lower) methyl α-L-fucoside in the presence of BC2L-C-nt (1000:1) recorded with a Bruker Avance 600 MHz spectrometer. The spectrum is recorded at 298K with irradiating frequency -0.05 ppm. In the STD spectrum, the signals of the fucose ring at 3.7 ppm and of the methyl group at 1.1 ppm are highlighted with red circles.

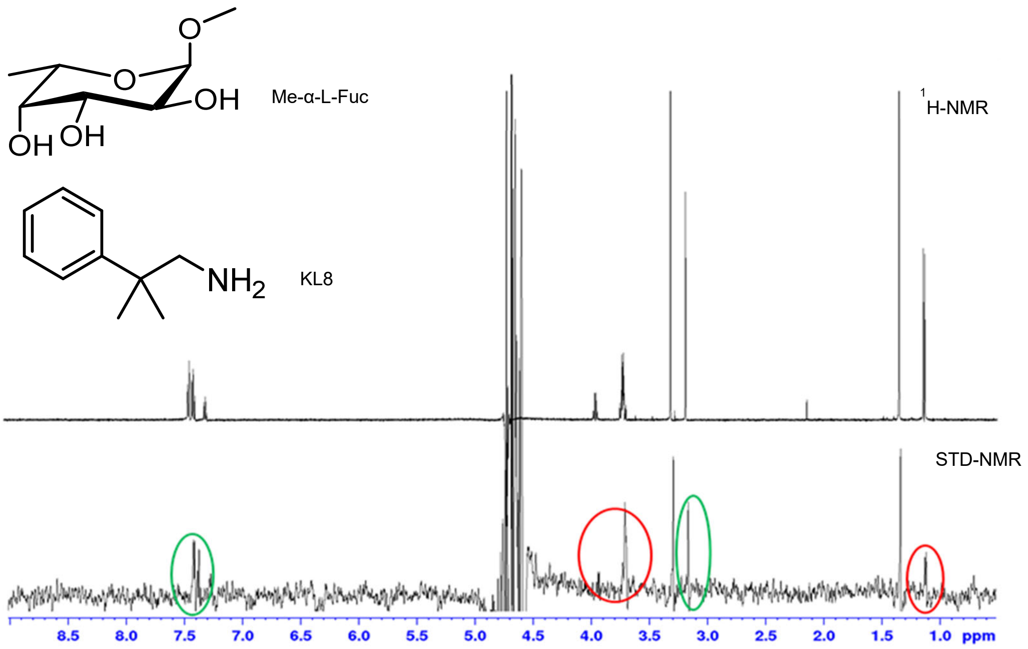

Figure S6 ^{1}H-NMR (upper) and STD spectrum (lower) of fragment KL8 and methyl α-L-fucoside in the presence of BC2L-C-nt (1000:1) recorded with a Bruker Avance 600 MHz spectrometer. The spectrum is recorded at 298K with the irradiating frequency at -0.05 ppm. In STD spectrum, the signals of the fucose ring around 3.7 ppm and of the methyl group at 1.1 ppm are highlighted with red circles. The signals of fragment KL8 are highlighted with a green circle (at 3.2 ppm for -CH2-NH2 and 7.4 ppm for aromatic protons).

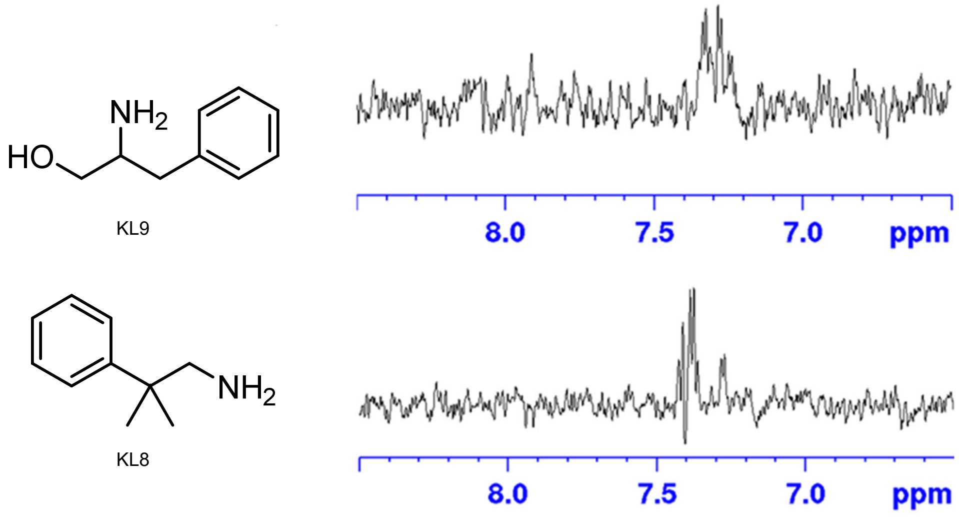

Figure S7 The STD spectra of fragment KL8 (lower) and KL9 (upper) in the presence of the BC2L-C-nt (1000:1) recorded in the presence of Methyl α-L-Fucoside with a Bruker Avance 600 MHz spectrometer. The spectra are recorded at 298K with the irradiating frequency at 10 ppm. In these spectra only the aromatic protons of the fragments are observable.

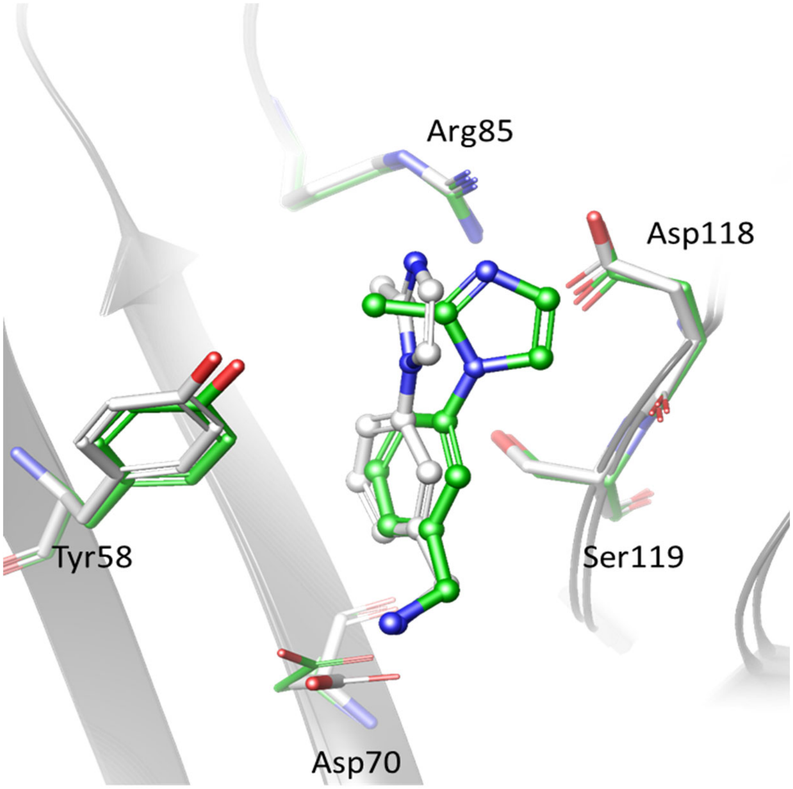

Figure S8 Comparison of binding pose of docked (grey) and crystallized complex (green) with KL3 (RMSD 0.4 Å).

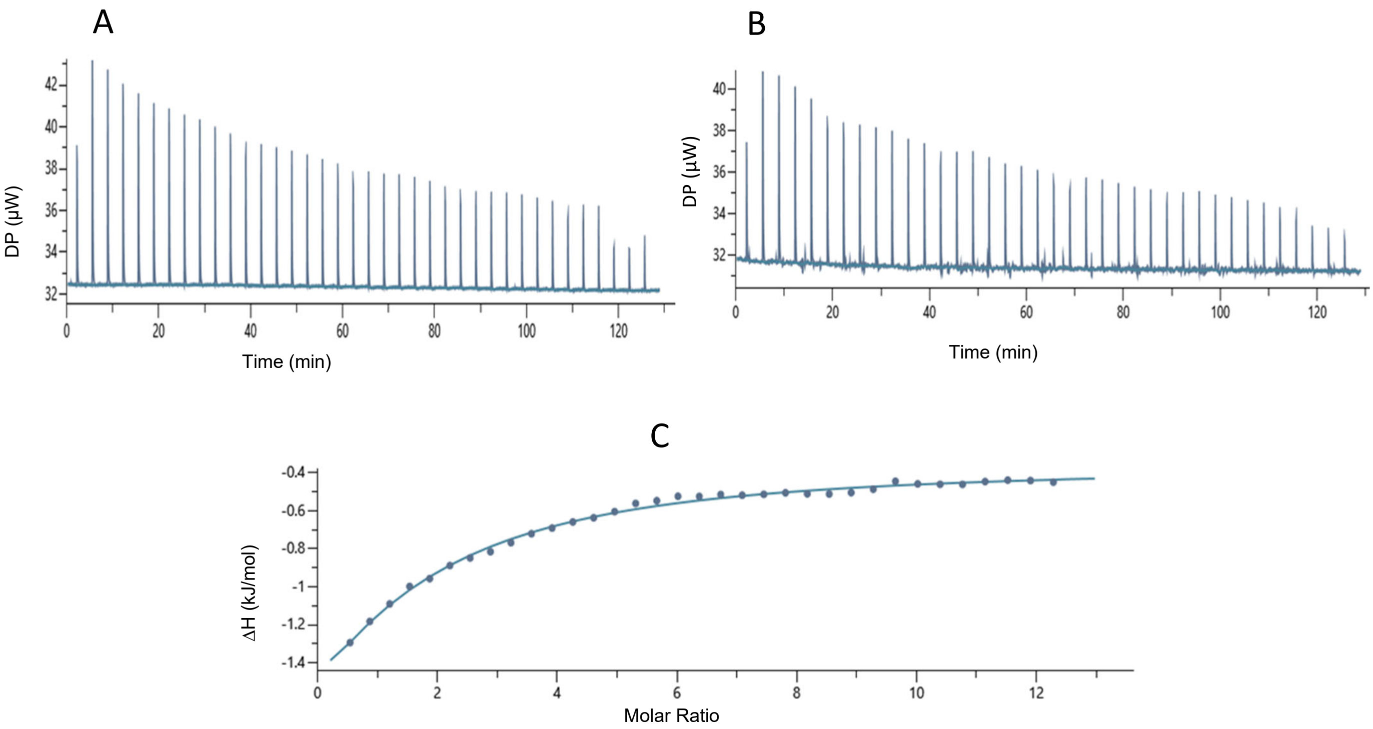

Figure S9 Isothermal microcalorimetry data. Titration of buffer by ligand (KL3, 15 mM) (A) and titration of BC2L-C-nt (225 µM) by KL3 (15 mM) at 25 °C (B). Point-by-point differences between ligand-in-protein and ligand-in-buffer for KL3 (C). The curve was fitted using the “one binding site” model.
